# How loss of tooth structure impacts the biomechanical behavior of a single-rooted maxillary premolar: FEA

**DOI:** 10.1007/s10266-023-00829-6

**Published:** 2023-07-02

**Authors:** Roaa Abdelwahab Abdelfattah, Nawar Naguib Nawar, Engy M. Kataia, Shehabeldin Mohamed Saber

**Affiliations:** 1 Department of Endodontics, Faculty of Dentistry, The British University in Egypt, Misr-Ismalia Road, El Sherouk City, Cairo, 11837 Egypt; 2 Department of Endodontics, Ain Shams University, Cairo, Egypt; 3 Centre for Innovative Dental Sciences (CIDS), Faculty of Dentistry, The British University in Egypt (BUE), El Sherouk City, 11837 Egypt

**Keywords:** Access cavity design, Different canal preparation, Finite element analysis

## Abstract

To evaluate the influence of the loss of coronal and radicular tooth structure on the biomechanical behavior and fatigue life of an endodontically treated maxillary premolar with confluent root canals using finite element analysis (FEA). An extracted maxillary second premolar was scanned to produce intact (IT) 3D model. Models were designed with an occlusal conservative access cavity (CAC) with different coronal defects; mesial defect (MO CAC), occlusal, mesial and distal defect (MOD CAC), and 2 different root canal preparations (30/.04, and 40/.04) producing 6 experimental models. FEA was used to study each model. A simulation of cycling loading of 50N was applied occlusally to stimulate the normal masticatory force. Number of cycles till failure (NCF) was used to compare strength of different models and stress distribution patterns via von Mises (vM) and maximum principal stress (MPS). The IT model survived 1.5 × 10^10^ cycles before failure, the CAC-30.04 had the longest survival of 1.59 × 10^9^, while the MOD CAC-40.04 had the shortest survival of 8.35 × 10^7^ cycles till failure. vM stress analysis showed that stress magnitudes were impacted by the progressive loss of coronal tooth structure rather than the radicular structure. MPS analysis showed that significant loss of coronal tooth structure translates into more tensile stresses. Given the limited size of maxillary premolars, marginal ridges have a critical role in the biomechanical behavior of the tooth. Access cavity preparation has a much bigger impact than radicular preparation on their strength and life span.

## Introduction

The biomechanical behavior of endodontically treated teeth (ETT) is affected by the amount of lost tooth structure before, during, and after treatment [1]. Loss of tooth integrity due to caries and extensive cavity preparation is the main reason for the reduced stiffness of ETT, rather than dehydration or physical changes in dentin [2, 3]. In addition, the lack of vitality severely limits sensory feedback during peak loads and makes non vital teeth more susceptible to fracture [4]. Unfortunately, most fractures are non-restorable [5].

Maxillary premolars have a high incidence [6] and greatest susceptibility [7] to fracture under occlusal loading. Their narrow cervical thickness, presence of a concavity on the mesial aspect of the root, and a radicular groove on the palatal aspect of the buccal root predispose them to cusp fractures, wedging, and splitting [8–10]_._

Previous experimental studies showed that loss of tooth walls, particularly the marginal ridges, causes a significant reduction in tooth fracture resistance more than different access cavity designs [11–14]_._ Nonetheless, the current endodontic literature lacks studies examining the interaction between coronal integrity, access designs, and shaping parameters on the biomechanical behavior and fatigue life of ETT.

This study sought to assess the influence of loss of the mesial, or the mesial and distal walls in combination with different access designs and shaping parameters on the biomechanical behavior and fatigue life of an endodontically treated maxillary second premolar with confluent root canals using finite element analysis (FEA) method.

FEA can isolate the effect of each parameter in a scenario that mimics the clinical situation [15–17]. Also, it overcomes the limitation of experimental laboratory tests, such as the standardization of teeth because of possible variations in dentin mechanical properties, age, tooth extraction forces, storage time, and storage medium after extraction [18]. The null hypothesis was that there is no difference between the effects of loss of coronal or radicular tooth structure on the biomechanical behavior and fatigue life of ETT.

## Materials and methods

### Finite element model generation

An intact, single-rooted maxillary second premolar with confluent canals and a mature apex was scanned using high-resolution Cone Beam Computed Tomography machine (Planmeca ProMax 3d MID; Planmeca, Helsinki, Finland) at 90 kV, 12 mA with a voxel dimension of 75 μm. Then, the generated DICOM images were 3D reconstructed using a Materialize interactive medical image control system (MIMICS version 21; Materialise, Leuven, Belgium). The same software was used to identify enamel and dentin and produce the three-dimensional model by forming masks and automatically growing threshold regions. Data were then optimized using the 3-Matic Medical 11.0 software (Materialise, Leuven, Belgium). SolidWorks (Dassault Systemes, Paris, France) to combine enamel and dentin and establish the surrounding periodontal ligaments and the surrounding bone [15–17]_._ Model validation was done according to Nawar et al. [17].

### Ethical committee approval

This study has ethical clearance from the research ethics committee Faculty of Dentistry at the British University in Egypt (FD BUE REC 21-003).

### Cavity designs

**Fig. 1:**
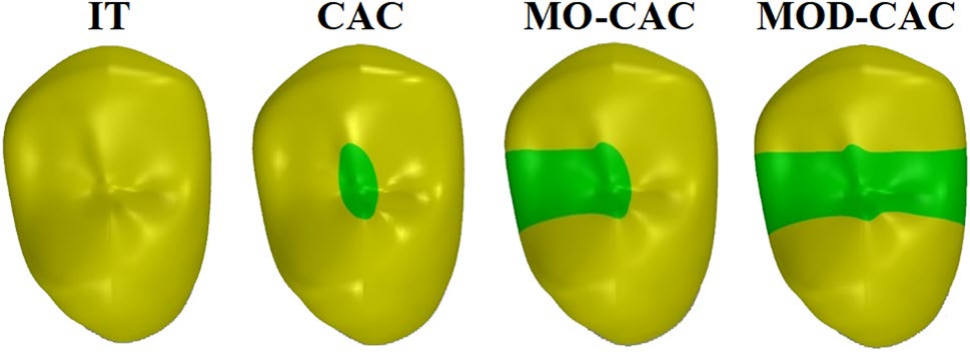
Occlusal view of simulated models with different cavity designs

**Fig. 2:**
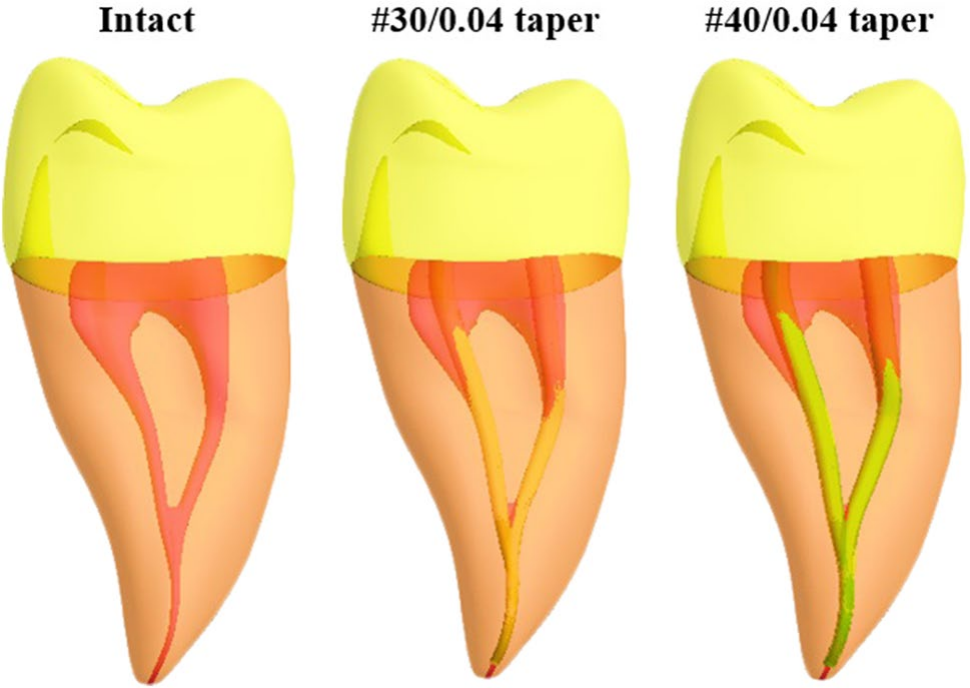
Root canal preparations were filled with simulated gutta-percha

After producing the intact (IT) model, 6 experimental models (3 coronal variations with 2 parameters for root canal shaping for each) were generated (Figs. 1 and 2);

A) Conservative Endodontic Access (CAC): designed by drawing lines from the center of each of the buccal and palatal root canal orifices at the furcation level, then extending them to the occlusal surface, resulting in 2 cross-points that were connected to produce the access outline [19, 20].
B) Conservative Endodontic Access along with loss of the mesial wall (MO CAC): To create the proximal part, the middle 1/3 of the buccopalatal width of the tooth in the mesial one third was removed including the marginal ridge with an occlusogingival depth of 1.5–2 mm on the outer surface [21, 22].
C) Conservative Endodontic Access with loss of both proximal walls (MOD CAC). Two proximal parts are created the same way done with the MO CAC.

The junctions between the occlusal and the proximal cavities were made to be smooth and curvy to avoid establishing stress concentration areas. Access cavities and missing proximal wall(s) were filled with simulated composite resin material (Fig. 1), and the volume of simulated composite representing the amount of lost tooth structure is listed in Table 1.

**Table 1:** The volume of simulated composite used to fill the experimental models in mm^3^

Model	Volume of simulated composite
CAC	22.32 mm^3^
MC CAC	51.83 mm^3^
MOD CAC	73.97 mm^3^

### Root canal preparation

Root canal preparations were simulated as either (#30/0.04 taper) or (#40/0.04 taper) as shaping parameters [16]_._ This was done by drawing a line in the central axis of the root canal, then creating a conical shape around it with the target dimensions. The prepared root canals were filled with simulated gutta-percha filling materials, 0.5 mm short from the apex of the root canal up to 2 mm from the canal orifices (Fig. 2).

### Meshing and set material properties

**Fig. 3:**
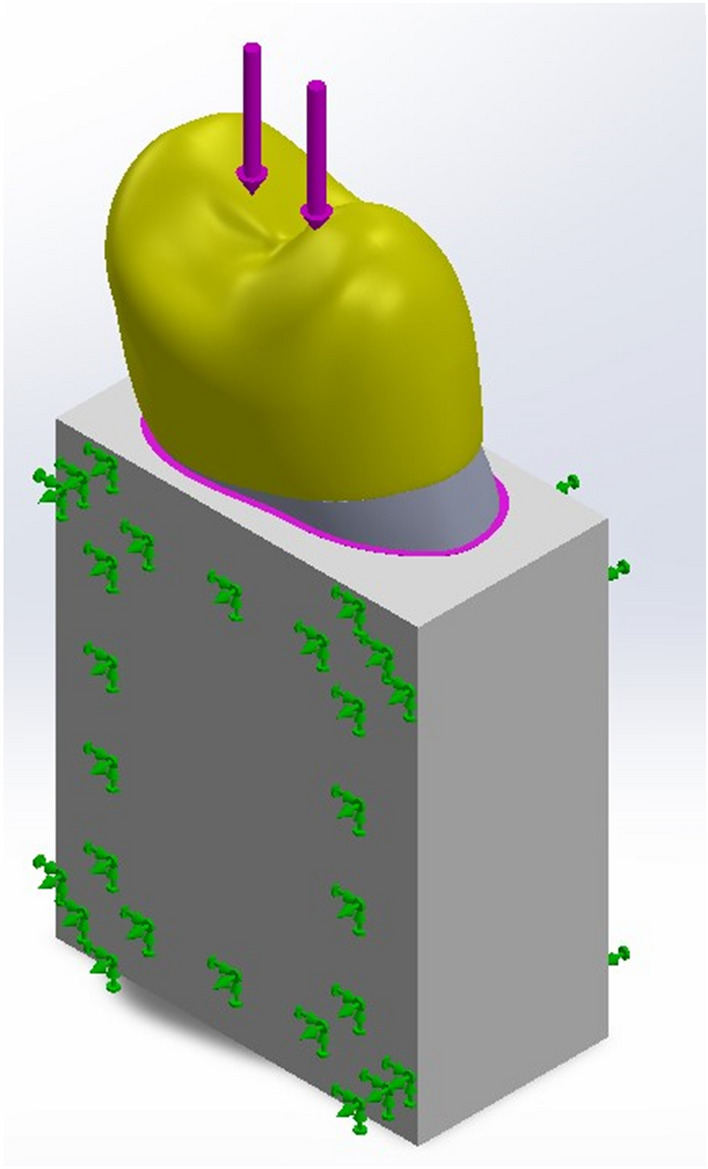
Simulated alveolar bone was constrained at mesial and distal aspects (green arrows). Models were subjected to cyclic occlusal loading with a magnitude of 50 N on the occlusal surface (vertical arrows on the occlusal surface)

All models were imported into the Cosmos software package (Solid works software package; Dassault Systems) for meshing. Teeth, as well as all materials used, were considered homogeneous, linear, and isotropic [23]. The elastic modulus and Poisson’s ratio of structures used to set up FEA models are listed in Table 2, whereas the plot of stress/Number of cycles to failure (SN curve) for both enamel and dentin was set according to Gao et al. [24] and Kinney et al. [25]. The numbers of nodes and tetrahedral elements ranged from 38,411 and 63,715, respectively (solid model), to 40,639 and 69,287, respectively (MOD CAC model). Considering the bounding conditions, the cancellous bone block was fixed mesially and distally and all components were simulated to have bonded contacts (Fig. 3).

**Table 2:** Mechanical Properties of the Materials for Finite Element Analysis (15–17)

Material	Young’s modulus (MPa)	Poisson’s ratio
Enamel	84,100	0.30
Dentin	18,600	0.31
Composite resin	7000	0.30
Gutta-percha	0.69	0.45
Periodontal ligament	68.9	0.45
Alveolar bone	13,700	0.30

### Finite element analysis

The models were subjected to cyclic loading with a magnitude of 50 N [26] to simulate the clinical masticatory loading. Loading areas used followed the pattern of Lim et al. [27]. First, the solid model loading simulation was done and the number of cycles until failure (NCF) was registered as well as the failure location. Other models’ simulations were then performed, and their life span was calculated as the percentage of the NCF of each model compared with the solid model. After load application of all models, mathematical analysis of the stress distribution patterns, von Mises (VM) stresses, and Maximum Principal Stress (MPS) were assessed using Cosmos software package (Solid works software package; Dassault Systems).

## Results

**Fig. 4:**
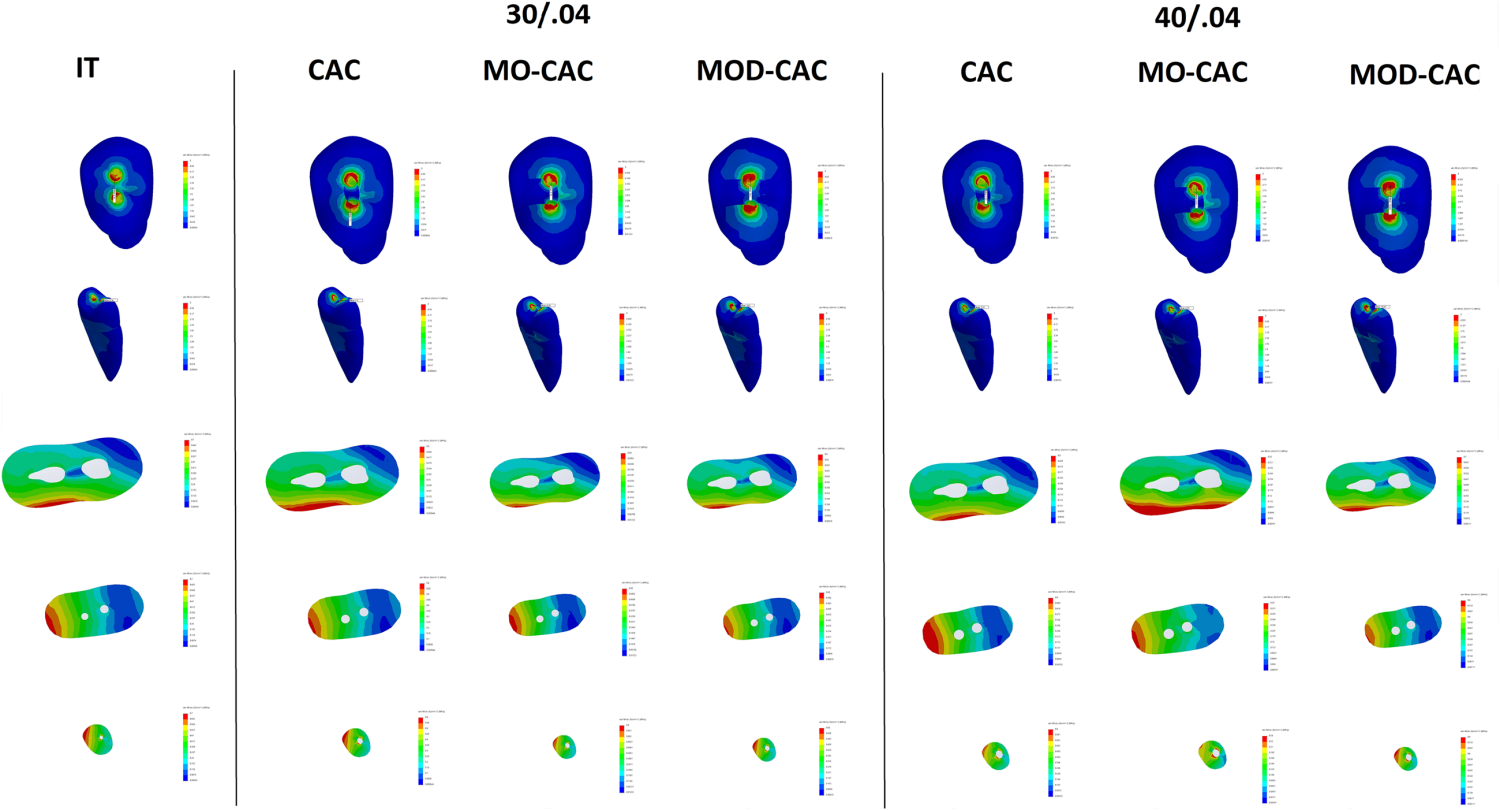
Composite figure showing the stress distribution of the VMS from different views (occlusal, isometric “mesio palatal line angle”, coronal cut at 12.5 mm from the apex, middle cut at 7 mm from the apex, and apical cut at 1.20 mm from the apex) of all models with different access cavities, and different root canal preparation

**Fig. 5:**
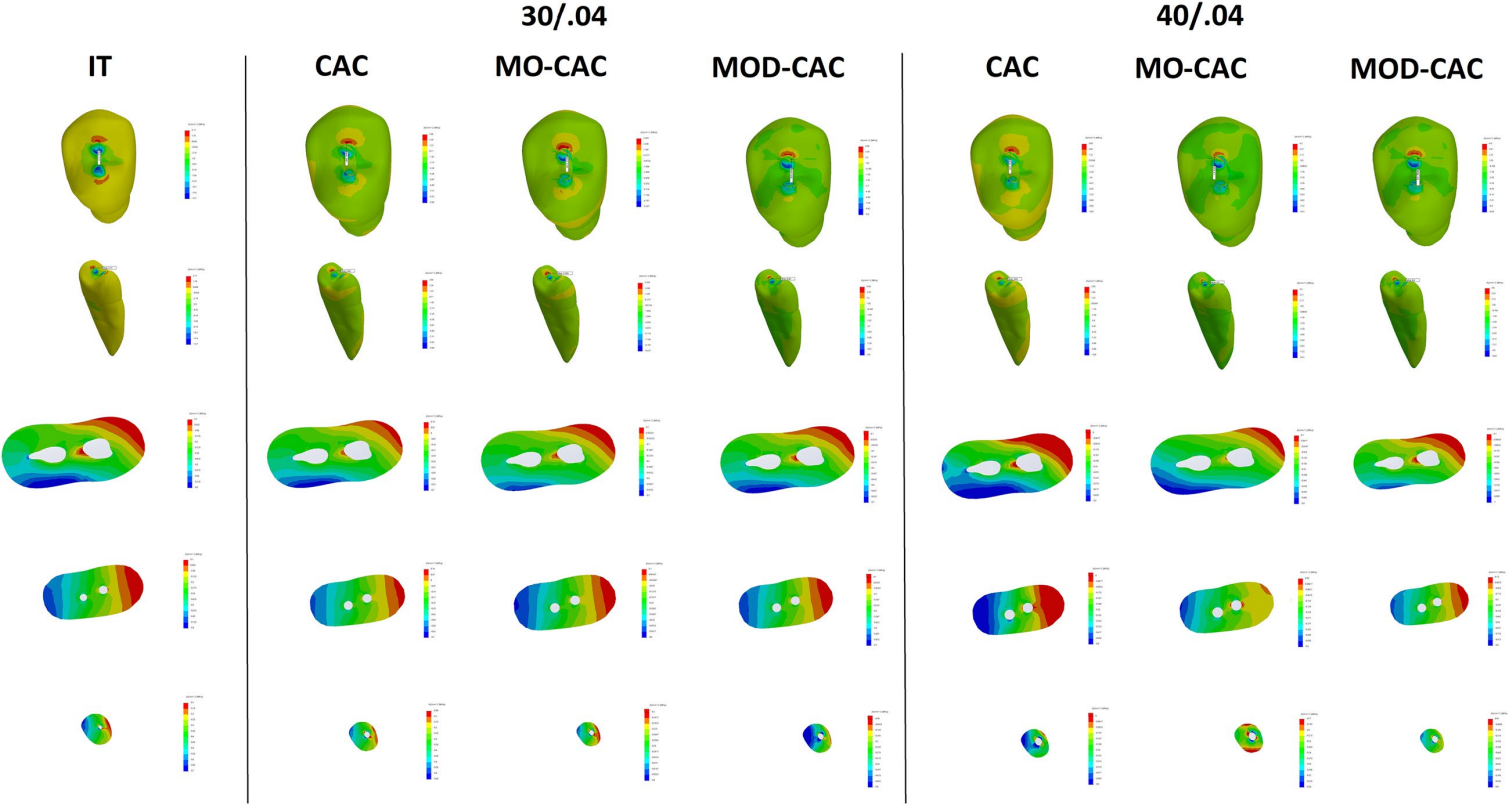
Composite figure showing the stress distribution of the MPS from different views (occlusal, isometric “mesio palatal line angle”, coronal cut at 12.5 mm from the apex, middle cut at 7 mm from the apex, and apical cut at 1.20 mm from the apex) of all models with different access cavities, and different root canal preparation

The increase (%) in stress within each model and the decrease (%) in the NCF compared with the IT model are presented in Table 3. Also, stress distribution patterns, maximum vM and MPS are shown in (Figs. 4 and 5).

**Table 3:** Maximum von Mises (VM) stress, number of cycles till failure (NCF), and the life span of various models compared to the intact model

Model	Canal preparation	Maximum VM stress (MPa)	NCF	Lifespan reduction (%)
Intact	N/A	6.14	1.50 × 10^10^	0.00%
CAC	30/0.04	7.70	1.59 × 10^9^	9.58%
	40/0.04	7.72	1.55 × 10^9^	9.69%
MO CAC	30/0.04	8.75	4.49 × 10^8^	14.97%
	40/0.04	8.78	4.36 × 10^8^	15.10%
MOD CAC	30/0.04	10.30	9.15 × 10^7^	21.76%
	40/0.04	10.37	8.35 × 10^7^	22.15%

The IT model had the least maximum vM stress with a value of 6.14 MPa and survived 1.5 × 10^10^ cycles before failure. CAC-30.04 had the longest survival of 1.59 × 10^9^ NCF and the least maximum vM of 7.7 MPa, while the MOD CAC-40.04 had the shortest survival of 8.35 × 10^7^ NCF and the highest maximum vM of 10.37 MPa.

Increasing the radicular shaping parameters from 30.04 to 40.04 had a minimal effect on vM stresses and NCF of all coronal configurations accounting for a lifespan reduction of 0.11% within the CAC models, 0.13% within the MO CAC models, and 0.39% within the MOD CAC models.

When the MO CAC and the MOD CAC models were compared together, the NCF ranged from 8.35 × 10^7^ for the MOD CAC-40.04 to 4.49 × 10^8^ for the MO CEC-30.04. Maximum vM stress ranged from 8.75 MPA with the MO CAC 30.04 model to 10.37 MPa for the MOD CAC 40.04.

**Fig. 6:**
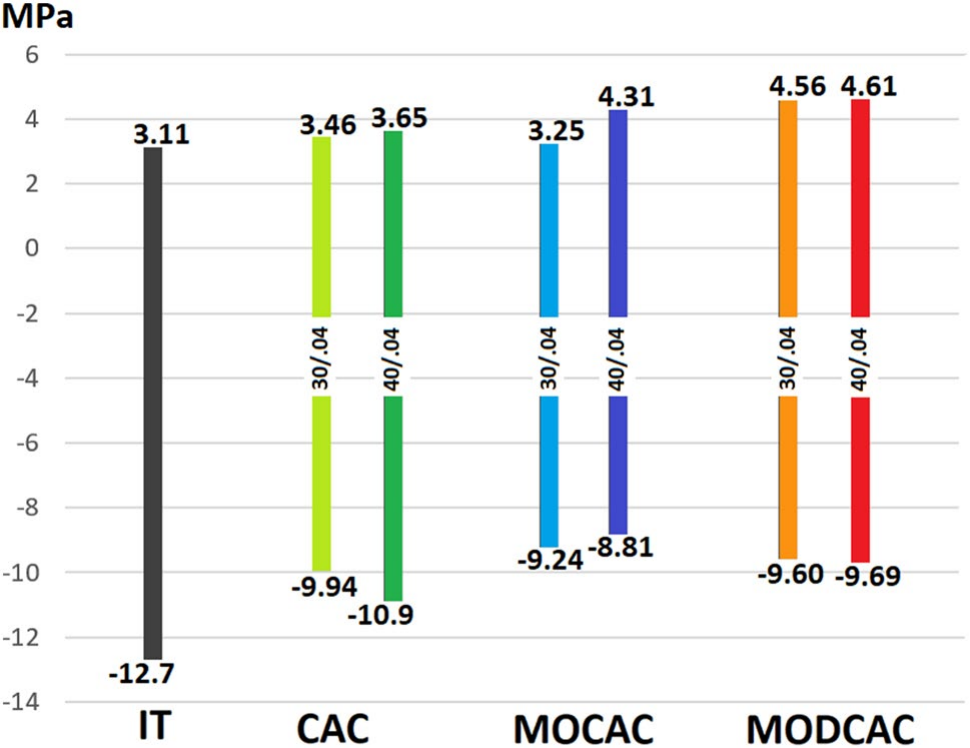
Bar chart showing MPS range in MPa (Negative sign denotes compressive stresses)

When MPS was analyzed, stress distribution patterns did not vary among experimental models with the maximum tensile stress located at the buccal restoration/tooth interface, however, stresses character leans more toward being tensile with the loss of more coronal structures. MPS values are displayed in Fig. 6.

## Discussion

Minimally invasive endodontics has been suggested to increase the life span of ETT through minimal dentin removal and preservation of the pericervical dentin [28]. This study investigated the impact of each of the loss of coronal and radicular tooth structure alone and combined on the life span of a tooth with a high fracture susceptibility.

FEA was used for stress analysis in the present study, because it has the merit of standardization through the evaluation of one tested variable while virtually fixating all other contributors, thus providing reliable results and numerically controlled testing [29]. Also, fracture methodology used for in vitro analyses of ETT does not accurately reflect intraoral conditions in which failures occur primarily because of fatigue [30].

The conservative access design was used in this study as previous reports demonstrated its superiority over the traditional access from the biomechanical perspective, whereas the ultraconservative access cavity designs such as the ninja access or orifice directed access were not included in this study, because the literature does not support the notion that they provide any additional mechanical advantage beyond what is provided by conservative designs [20]. On the other hand, evidence suggests that they compromise cleaning and predispose to procedural errors [31].

In this study, simulated cyclic loading was applied, given the fact that clinically most of the failures of ETT are caused by cyclic fatigue with a subcritical load or fluctuating stresses which are much lower than the load capacity required to cause a catastrophic failure [25]. Such repeated masticatory loading cycles cause fatigue failure due to the cumulative effect of crack initiation and propagation over time [32].

This study presented that the loss of a proximal wall had a considerable impact on the life span of ETT with a reduction of approximately 6% of the tooth’s expected life. Analyzing the maximum vM stress values demonstrated that the recorded stress magnitude is directly proportional to the amount of dental structure lost. When the CAC model is compared to the intact model, stresses are raised by approximately 25.4%. This stress increase reached 42.5% with the loss of the mesial marginal ridge in the MO CAC model, and 67.7% with the additional loss of the distal marginal ridge in the MOD CAC model. This agrees with Reeh et al. [2] and Corsentino et al. [12] who recommended the preservation of marginal ridge(s) integrity to maintain tooth strength.

Analysis of stresses whether in Maximum Principal stresses or von Mises stresses were reflected in the fatigue, the solid model was used as a control reference, and the CAC showed the highest life log percentage of 90.42% followed by the MC CAC with 85.03% and the least lifelog percentage was recorded for MOD CAC model by 78.24%. This comes in agreement with previous studies [15–17]. It is worth mentioning that the value of tensile stresses in the tooth with a MOD cavity (≈ 4.5 MPa) was approximately 50% more than that found in the intact tooth (3.1 MPa). This may be a concern given that dental tissues are more vulnerable to stresses with tensile character [33, 34] and justify adopting full coverage restorations in cases where both proximal sides of a maxillary premolar are compromised.

Finally, our study showed that varying the apical preparation of the root canals did not appear to make a substantial mechanical difference regardless of the amount of coronal structures lost. This agrees with previous studies [15–17] and emphasizes that most of the functional loads are absorbed and housed by the coronal structures. This finding contrasts with Smoljan et al. [35] who found that wider progressive taper preparations have less fracture resistance than narrow progressive taper canal preparations. This can be attributed to the difference in loading conditions as they utilized 200N in static loading and the numerical values of total deformation as an indicator of fracture [33].

Most of the limitations of this study are linked to FEA, which is a computerized virtual method that differs from the clinical scenario that cannot be easily replicated. The mechanical properties of materials are set as uniform isotropic materials, when in fact it is well established that dental structures including the tubular structure of dentin and dentin-enamel junction are functionally graded materials with varying elastic models and creep-related behavior [17].

## Conclusion

1. The biomechanical behavior and the life span of maxillary premolars were more influenced by the loss of coronal tooth structure.
2. The marginal ridges are structurally very important and should be preserved whenever possible.
3. As long as the extent of the radicular preparation is kept within the widely accepted preparation sizes, it has almost no effect on the fatigue life of endodontically treated single-rooted maxillary premolars.

## Data Availability

The datasets used and/or analyzed during the current study are available from the corresponding author on reasonable request.
